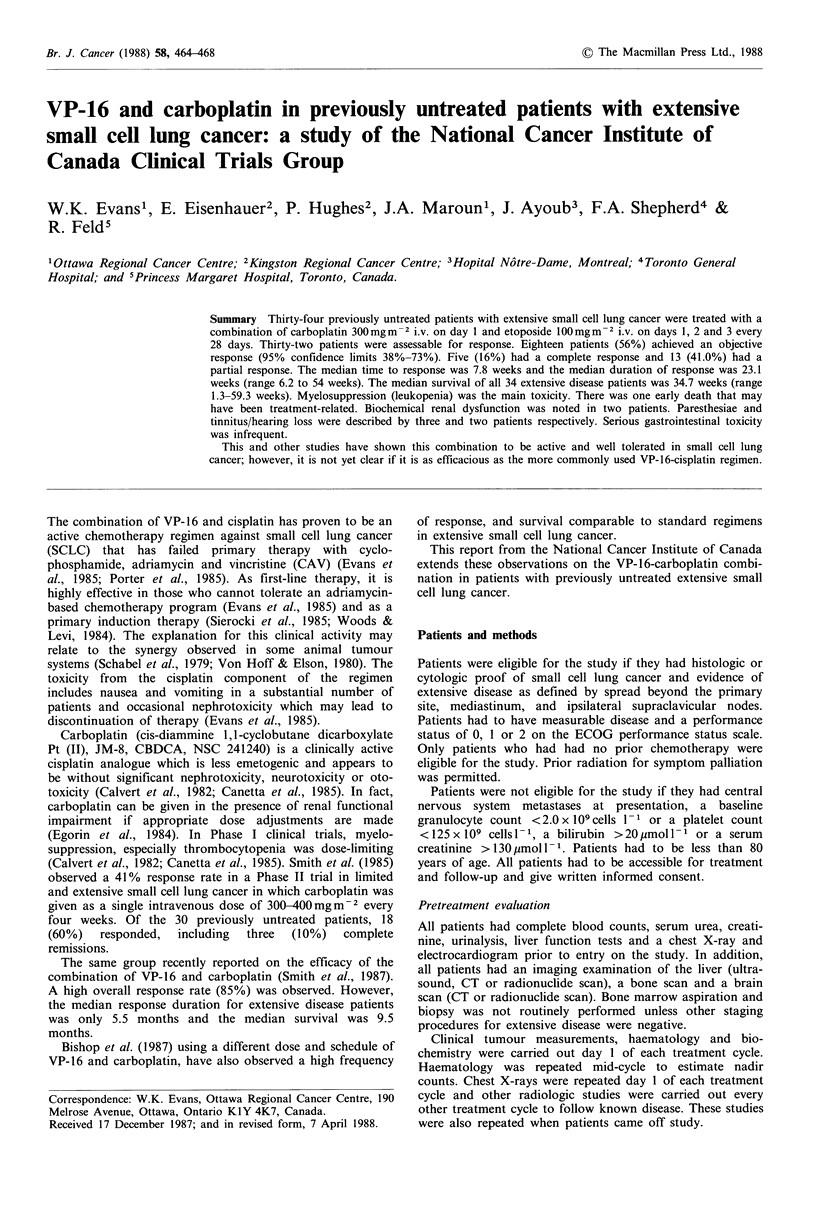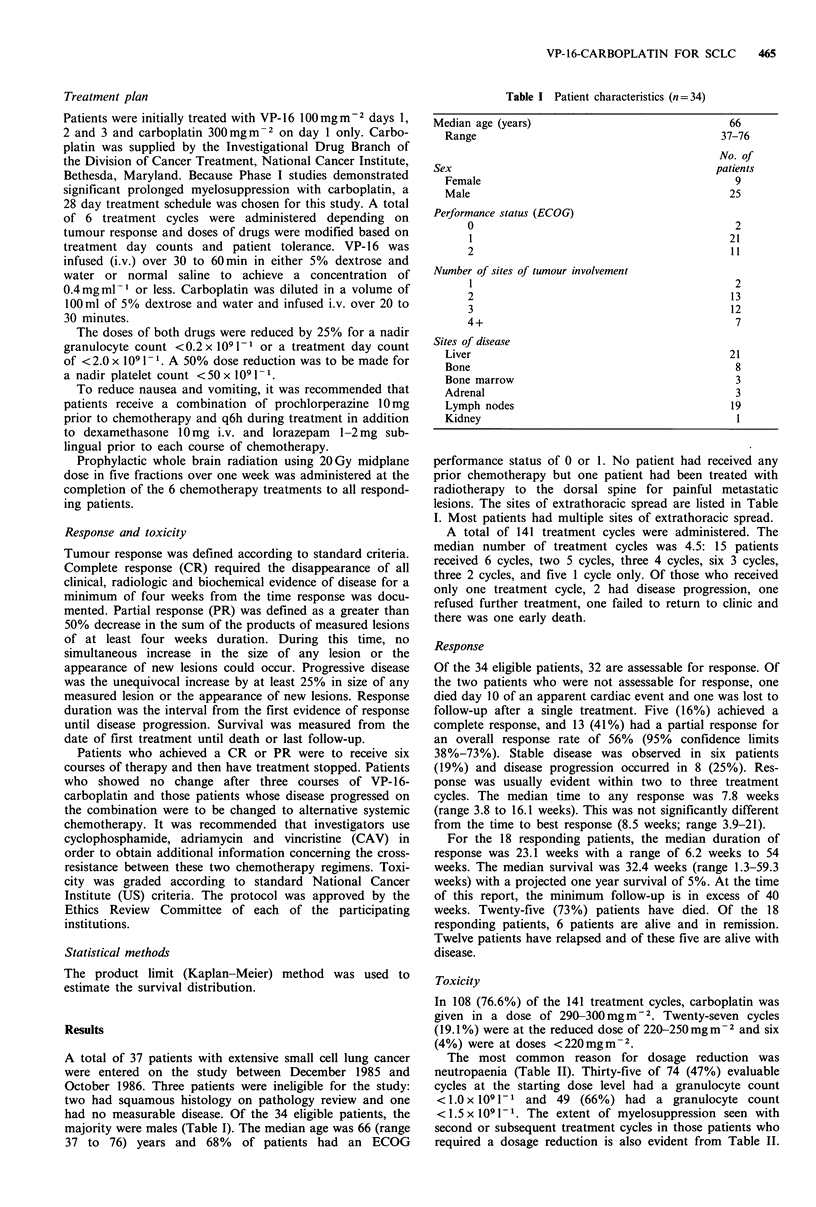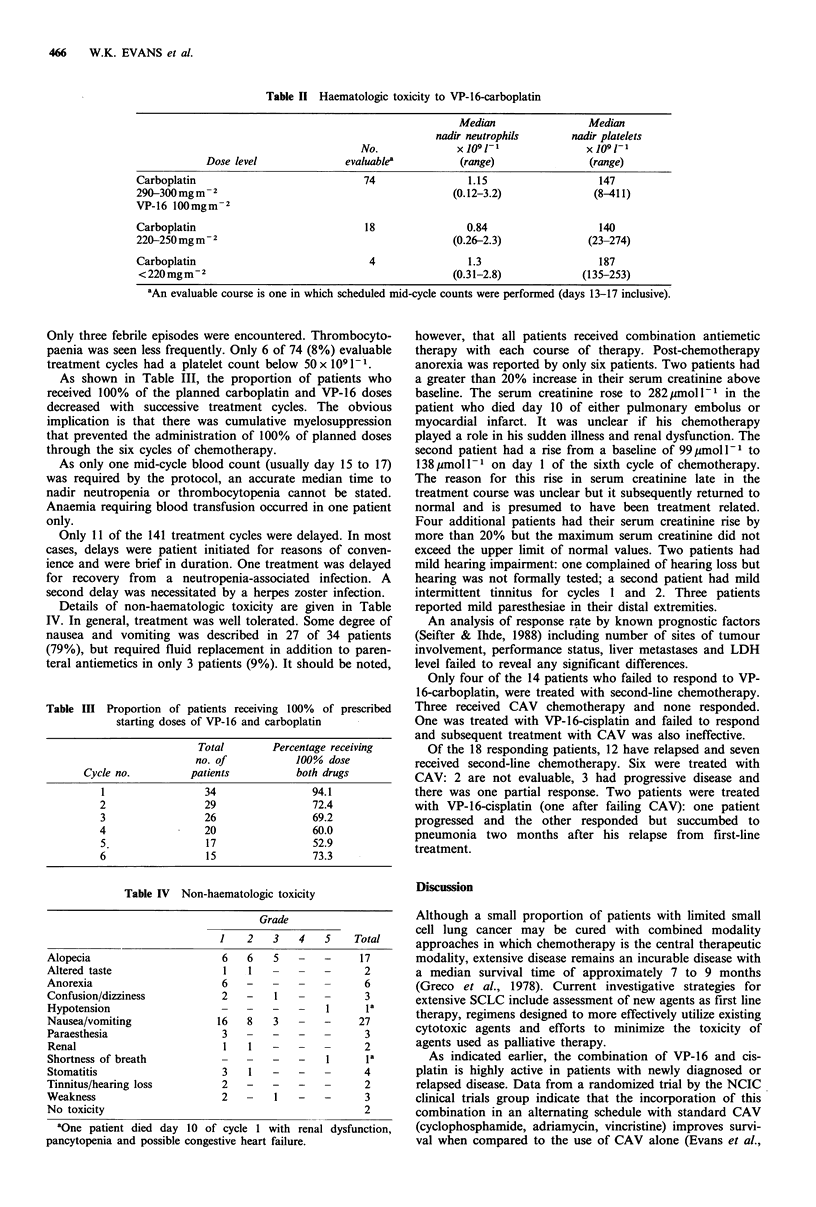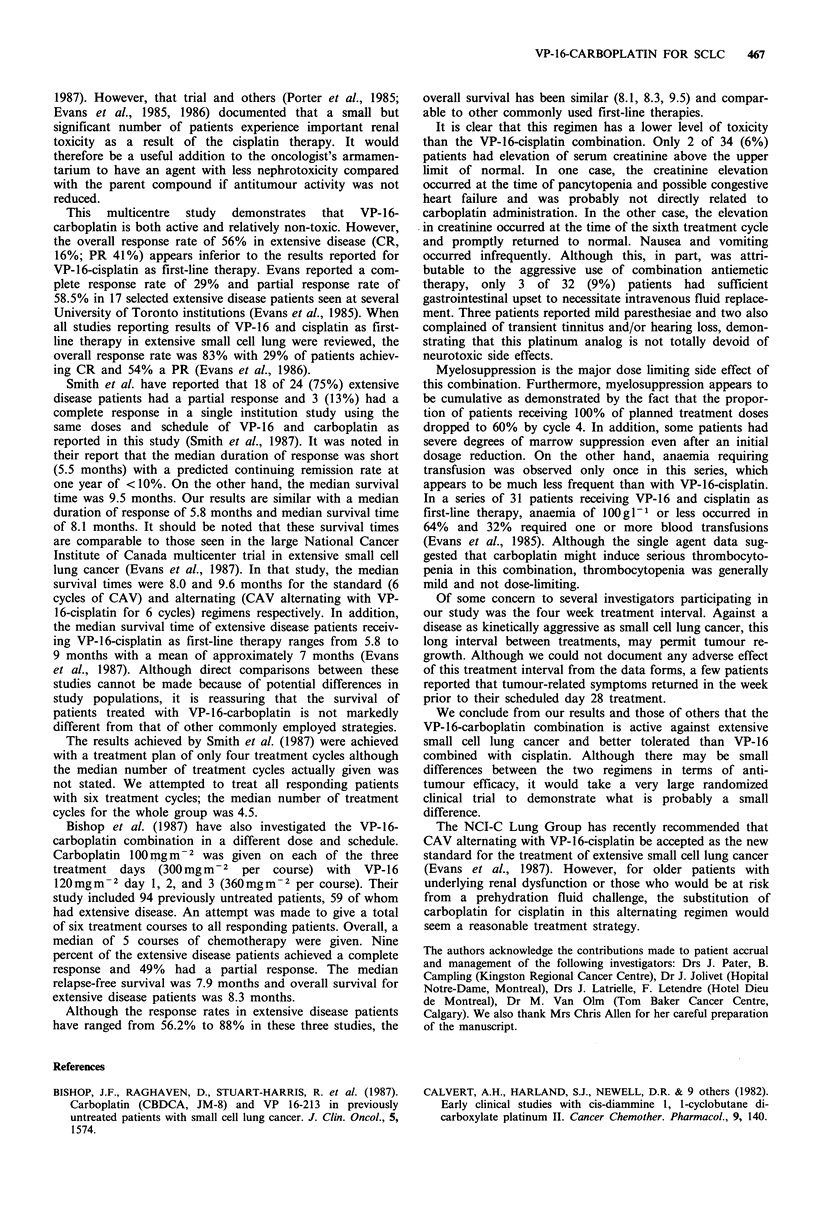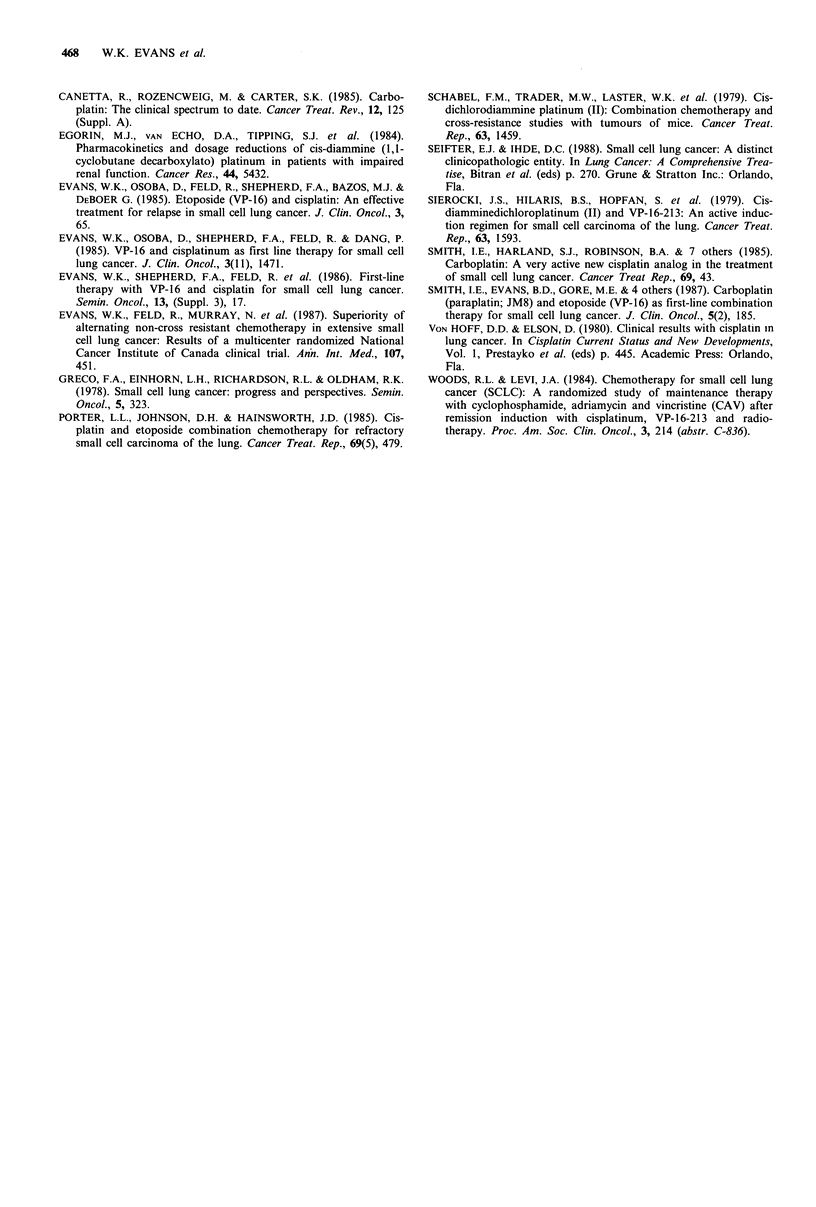# VP-16 and carboplatin in previously untreated patients with extensive small cell lung cancer: a study of the National Cancer Institute of Canada Clinical Trials Group.

**DOI:** 10.1038/bjc.1988.242

**Published:** 1988-10

**Authors:** W. K. Evans, E. Eisenhauer, P. Hughes, J. A. Maroun, J. Ayoub, F. A. Shepherd, R. Feld

**Affiliations:** Ottawa Regional Cancer Centre, Canada.

## Abstract

Thirty-four previously untreated patients with extensive small cell lung cancer were treated with a combination of carboplatin 300 mg m-2 i.v. on day 1 and etoposide 100 mg m-2 i.v. on days 1, 2 and 3 every 28 days. Thirty-two patients were assessable for response. Eighteen patients (56%) achieved an objective response (95% confidence limits 38%-73%). Five (16%) had a complete response and 13 (41.0%) had a partial response. The median time to response was 7.8 weeks and the median duration of response was 23.1 weeks (range 6.2 to 54 weeks). The median survival of all 34 extensive disease patients was 34.7 weeks (range 1.3-59.3 weeks). Myelosuppression (leukopenia) was the main toxicity. There was one early death that may have been treatment-related. Biochemical renal dysfunction was noted in two patients. Paresthesiae and tinnitus/hearing loss were described by three and two patients respectively. Serious gastrointestinal toxicity was infrequent. This and other studies have shown this combination to be active and well tolerated in small cell lung cancer; however, it is not yet clear if it is as efficacious as the more commonly used VP-16-cisplatin regimen.


Br. J. Cancer (1988) 58, 464–468 © The Macmillan Press Ltd., 1988

### VP-16 and carboplatin in previously untreated patients with extensive small cell lung cancer: a study of the National Cancer Institute of Canada Clinical Trials Group

W.K. Evans[1], E. Eisenhauer[2], P. Hughes[2], J.A. Maroun[1], J. Ayoub[3], F.A. Shepherd[4] & R. Feld[5]

[1]*Ottawa Regional Cancer Centre;* [2]*Kingston Regional Cancer Centre;* [3]*Hopital Nôtre-Dame, Montreal;* [4]*Toronto General Hospital; and* [5]*Princess Margaret Hospital, Toronto, Canada.*

**Summary** Thirty-four previously untreated patients with extensive small cell lung cancer were treated with a combination of carboplatin 300 mg m$^{-2}$ i.v. on day 1 and etoposide 100 mg m$^{-2}$ i.v. on days 1, 2 and 3 every 28 days. Thirty-two patients were assessable for response. Eighteen patients (56%) achieved an objective response (95% confidence limits 38%–73%). Five (16%) had a complete response and 13 (41.0%) had a partial response. The median time to response was 7.8 weeks and the median duration of response was 23.1 weeks (range 6.2 to 54 weeks). The median survival of all 34 extensive disease patients was 34.7 weeks (range 1.3–59.3 weeks). Myelosuppression (leukopenia) was the main toxicity. There was one early death that may have been treatment-related. Biochemical renal dysfunction was noted in two patients. Paresthesiae and tinnitus/hearing loss were described by three and two patients respectively. Serious gastrointestinal toxicity was infrequent.

This and other studies have shown this combination to be active and well tolerated in small cell lung cancer; however, it is not yet clear if it is as efficacious as the more commonly used VP-16-cisplatin regimen.

The combination of VP-16 and cisplatin has proven to be an active chemotherapy regimen against small cell lung cancer (SCLC) that has failed primary therapy with cyclophosphamide, adriamycin and vincristine (CAV) (Evans *et al.*, 1985; Porter *et al.*, 1985). As first-line therapy, it is highly effective in those who cannot tolerate an adriamycin-based chemotherapy program (Evans *et al.*, 1985) and as a primary induction therapy (Sierocki *et al.*, 1985; Woods & Levi, 1984). The explanation for this clinical activity may relate to the synergy observed in some animal tumour systems (Schabel *et al.*, 1979; Von Hoff & Elson, 1980). The toxicity from the cisplatin component of the regimen includes nausea and vomiting in a substantial number of patients and occasional nephrotoxicity which may lead to discontinuation of therapy (Evans *et al.*, 1985).

Carboplatin (cis-diammine 1,1-cyclobutane dicarboxylate Pt (II), JM-8, CBDCA, NSC 241240) is a clinically active cisplatin analogue which is less emetogenic and appears to be without significant nephrotoxicity, neurotoxicity or ototoxicity (Calvert *et al.*, 1982; Canetta *et al.*, 1985). In fact, carboplatin can be given in the presence of renal functional impairment if appropriate dose adjustments are made (Egorin *et al.*, 1984). In Phase I clinical trials, myelosuppression, especially thrombocytopenia was dose-limiting (Calvert *et al.*, 1982; Canetta *et al.*, 1985). Smith *et al.* (1985) observed a 41% response rate in a Phase II trial in limited and extensive small cell lung cancer in which carboplatin was given as a single intravenous dose of 300–400 mg m$^{-2}$ every four weeks. Of the 30 previously untreated patients, 18 (60%) responded, including three (10%) complete remissions.

The same group recently reported on the efficacy of the combination of VP-16 and carboplatin (Smith *et al.*, 1987). A high overall response rate (85%) was observed. However, the median response duration for extensive disease patients was only 5.5 months and the median survival was 9.5 months.

Bishop *et al.* (1987) using a different dose and schedule of VP-16 and carboplatin, have also observed a high frequency of response, and survival comparable to standard regimens in extensive small cell lung cancer.

This report from the National Cancer Institute of Canada extends these observations on the VP-16-carboplatin combination in patients with previously untreated extensive small cell lung cancer.

### Patients and methods

Patients were eligible for the study if they had histologic or cytologic proof of small cell lung cancer and evidence of extensive disease as defined by spread beyond the primary site, mediastinum, and ipsilateral supraclavicular nodes. Patients had to have measurable disease and a performance status of 0, 1 or 2 on the ECOG performance status scale. Only patients who had had no prior chemotherapy were eligible for the study. Prior radiation for symptom palliation was permitted.

Patients were not eligible for the study if they had central nervous system metastases at presentation, a baseline granulocyte count $<2.0\times10^9$ cells l$^{-1}$ or a platelet count $<125\times10^9$ cells l$^{-1}$, a bilirubin $>20\,\mu$mol l$^{-1}$ or a serum creatinine $>130\,\mu$mol l$^{-1}$. Patients had to be less than 80 years of age. All patients had to be accessible for treatment and follow-up and give written informed consent.

### *Pretreatment evaluation*

All patients had complete blood counts, serum urea, creatinine, urinalysis, liver function tests and a chest X-ray and electrocardiogram prior to entry on the study. In addition, all patients had an imaging examination of the liver (ultrasound, CT or radionuclide scan), a bone scan and a brain scan (CT or radionuclide scan). Bone marrow aspiration and biopsy was not routinely performed unless other staging procedures for extensive disease were negative.

Clinical tumour measurements, haematology and biochemistry were carried out day 1 of each treatment cycle. Haematology was repeated mid-cycle to estimate nadir counts. Chest X-rays were repeated day 1 of each treatment cycle and other radiologic studies were carried out every other treatment cycle to follow known disease. These studies were also repeated when patients came off study.

Correspondence: W.K. Evans, Ottawa Regional Cancer Centre, 190 Melrose Avenue, Ottawa, Ontario K1Y 4K7, Canada.
Received 17 December 1987; and in revised form, 7 April 1988.

*Treatment plan*

Patients were initially treated with VP-16 $100\,mg\,m^{-2}$ days 1, 2 and 3 and carboplatin $300\,mg\,m^{-2}$ on day 1 only. Carboplatin was supplied by the Investigational Drug Branch of the Division of Cancer Treatment, National Cancer Institute, Bethesda, Maryland. Because Phase I studies demonstrated significant prolonged myelosuppression with carboplatin, a 28 day treatment schedule was chosen for this study. A total of 6 treatment cycles were administered depending on tumour response and doses of drugs were modified based on treatment day counts and patient tolerance. VP-16 was infused (i.v.) over 30 to 60 min in either 5% dextrose and water or normal saline to achieve a concentration of $0.4\,mg\,ml^{-1}$ or less. Carboplatin was diluted in a volume of 100 ml of 5% dextrose and water and infused i.v. over 20 to 30 minutes.

The doses of both drugs were reduced by 25% for a nadir granulocyte count $<0.2\times10^9\,l^{-1}$ or a treatment day count of $<2.0\times10^9\,l^{-1}$. A 50% dose reduction was to be made for a nadir platelet count $<50\times10^9\,l^{-1}$.

To reduce nausea and vomiting, it was recommended that patients receive a combination of prochlorperazine 10 mg prior to chemotherapy and q6h during treatment in addition to dexamethasone 10 mg i.v. and lorazepam 1–2 mg sublingual prior to each course of chemotherapy.

Prophylactic whole brain radiation using 20 Gy midplane dose in five fractions over one week was administered at the completion of the 6 chemotherapy treatments to all responding patients.

*Response and toxicity*

Tumour response was defined according to standard criteria. Complete response (CR) required the disappearance of all clinical, radiologic and biochemical evidence of disease for a minimum of four weeks from the time response was documented. Partial response (PR) was defined as a greater than 50% decrease in the sum of the products of measured lesions of at least four weeks duration. During this time, no simultaneous increase in the size of any lesion or the appearance of new lesions could occur. Progressive disease was the unequivocal increase by at least 25% in size of any measured lesion or the appearance of new lesions. Response duration was the interval from the first evidence of response until disease progression. Survival was measured from the date of first treatment until death or last follow-up.

Patients who achieved a CR or PR were to receive six courses of therapy and then have treatment stopped. Patients who showed no change after three courses of VP-16-carboplatin and those patients whose disease progressed on the combination were to be changed to alternative systemic chemotherapy. It was recommended that investigators use cyclophosphamide, adriamycin and vincristine (CAV) in order to obtain additional information concerning the cross-resistance between these two chemotherapy regimens. Toxicity was graded according to standard National Cancer Institute (US) criteria. The protocol was approved by the Ethics Review Committee of each of the participating institutions.

*Statistical methods*

The product limit (Kaplan–Meier) method was used to estimate the survival distribution.

### Results

A total of 37 patients with extensive small cell lung cancer were entered on the study between December 1985 and October 1986. Three patients were ineligible for the study: two had squamous histology on pathology review and one had no measurable disease. Of the 34 eligible patients, the majority were males (Table I). The median age was 66 (range 37 to 76) years and 68% of patients had an ECOG performance status of 0 or 1. No patient had received any prior chemotherapy but one patient had been treated with radiotherapy to the dorsal spine for painful metastatic lesions. The sites of extrathoracic spread are listed in Table I. Most patients had multiple sites of extrathoracic spread.

**Table I** Patient characteristics ($n=34$)

| | |
|---|---|
| Median age (years) | 66 |
| Range | 37–76 |
| *Sex* | *No. of patients* |
| Female | 9 |
| Male | 25 |
| *Performance status (ECOG)* | |
| 0 | 2 |
| 1 | 21 |
| 2 | 11 |
| *Number of sites of tumour involvement* | |
| 1 | 2 |
| 2 | 13 |
| 3 | 12 |
| 4+ | 7 |
| *Sites of disease* | |
| Liver | 21 |
| Bone | 8 |
| Bone marrow | 3 |
| Adrenal | 3 |
| Lymph nodes | 19 |
| Kidney | 1 |

A total of 141 treatment cycles were administered. The median number of treatment cycles was 4.5: 15 patients received 6 cycles, two 5 cycles, three 4 cycles, six 3 cycles, three 2 cycles, and five 1 cycle only. Of those who received only one treatment cycle, 2 had disease progression, one refused further treatment, one failed to return to clinic and there was one early death.

*Response*

Of the 34 eligible patients, 32 are assessable for response. Of the two patients who were not assessable for response, one died day 10 of an apparent cardiac event and one was lost to follow-up after a single treatment. Five (16%) achieved a complete response, and 13 (41%) had a partial response for an overall response rate of 56% (95% confidence limits 38%–73%). Stable disease was observed in six patients (19%) and disease progression occurred in 8 (25%). Response was usually evident within two to three treatment cycles. The median time to any response was 7.8 weeks (range 3.8 to 16.1 weeks). This was not significantly different from the time to best response (8.5 weeks; range 3.9–21).

For the 18 responding patients, the median duration of response was 23.1 weeks with a range of 6.2 weeks to 54 weeks. The median survival was 32.4 weeks (range 1.3–59.3 weeks) with a projected one year survival of 5%. At the time of this report, the minimum follow-up is in excess of 40 weeks. Twenty-five (73%) patients have died. Of the 18 responding patients, 6 patients are alive and in remission. Twelve patients have relapsed and of these five are alive with disease.

*Toxicity*

In 108 (76.6%) of the 141 treatment cycles, carboplatin was given in a dose of $290–300\,mg\,m^{-2}$. Twenty-seven cycles (19.1%) were at the reduced dose of $220–250\,mg\,m^{-2}$ and six (4%) were at doses $<220\,mg\,m^{-2}$.

The most common reason for dosage reduction was neutropaenia (Table II). Thirty-five of 74 (47%) evaluable cycles at the starting dose level had a granulocyte count $<1.0\times10^9\,l^{-1}$ and 49 (66%) had a granulocyte count $<1.5\times10^9\,l^{-1}$. The extent of myelosuppression seen with second or subsequent treatment cycles in those patients who required a dosage reduction is also evident from Table II.

**Table II** Haematologic toxicity to VP-16-carboplatin

| Dose level | No. evaluable[a] | Median nadir neutrophils $\times 10^9 l^{-1}$ (range) | Median nadir platelets $\times 10^9 l^{-1}$ (range) |
|---|---|---|---|
| Carboplatin 290–300 mg m$^{-2}$ VP-16 100 mg m$^{-2}$ | 74 | 1.15 (0.12–3.2) | 147 (8–411) |
| Carboplatin 220–250 mg m$^{-2}$ | 18 | 0.84 (0.26–2.3) | 140 (23–274) |
| Carboplatin <220 mg m$^{-2}$ | 4 | 1.3 (0.31–2.8) | 187 (135–253) |

[a]An evaluable course is one in which scheduled mid-cycle counts were performed (days 13–17 inclusive).

Only three febrile episodes were encountered. Thrombocytopaenia was seen less frequently. Only 6 of 74 (8%) evaluable treatment cycles had a platelet count below $50 \times 10^9 l^{-1}$.

As shown in Table III, the proportion of patients who received 100% of the planned carboplatin and VP-16 doses decreased with successive treatment cycles. The obvious implication is that there was cumulative myelosuppression that prevented the administration of 100% of planned doses through the six cycles of chemotherapy.

As only one mid-cycle blood count (usually day 15 to 17) was required by the protocol, an accurate median time to nadir neutropenia or thrombocytopenia cannot be stated. Anaemia requiring blood transfusion occurred in one patient only.

Only 11 of the 141 treatment cycles were delayed. In most cases, delays were patient initiated for reasons of convenience and were brief in duration. One treatment was delayed for recovery from a neutropenia-associated infection. A second delay was necessitated by a herpes zoster infection.

Details of non-haematologic toxicity are given in Table IV. In general, treatment was well tolerated. Some degree of nausea and vomiting was described in 27 of 34 patients (79%), but required fluid replacement in addition to parenteral antiemetics in only 3 patients (9%). It should be noted,

**Table III** Proportion of patients receiving 100% of prescribed starting doses of VP-16 and carboplatin

| Cycle no. | Total no. of patients | Percentage receiving 100% dose both drugs |
|---|---|---|
| 1 | 34 | 94.1 |
| 2 | 29 | 72.4 |
| 3 | 26 | 69.2 |
| 4 | 20 | 60.0 |
| 5 | 17 | 52.9 |
| 6 | 15 | 73.3 |

**Table IV** Non-haematologic toxicity

| | Grade | | | | | |
|---|---|---|---|---|---|---|
| | 1 | 2 | 3 | 4 | 5 | Total |
| Alopecia | 6 | 6 | 5 | – | – | 17 |
| Altered taste | 1 | 1 | – | – | – | 2 |
| Anorexia | 6 | – | – | – | – | 6 |
| Confusion/dizziness | 2 | – | 1 | – | – | 3 |
| Hypotension | – | – | – | – | 1 | 1[a] |
| Nausea/vomiting | 16 | 8 | 3 | – | – | 27 |
| Paraesthesia | 3 | – | – | – | – | 3 |
| Renal | 1 | 1 | – | – | – | 2 |
| Shortness of breath | – | – | – | – | 1 | 1[a] |
| Stomatitis | 3 | 1 | – | – | – | 4 |
| Tinnitus/hearing loss | 2 | – | – | – | – | 2 |
| Weakness | 2 | – | 1 | – | – | 3 |
| No toxicity | | | | | | 2 |

[a]One patient died day 10 of cycle 1 with renal dysfunction, pancytopenia and possible congestive heart failure.

however, that all patients received combination antiemetic therapy with each course of therapy. Post-chemotherapy anorexia was reported by only six patients. Two patients had a greater than 20% increase in their serum creatinine above baseline. The serum creatinine rose to 282 $\mu$mol l$^{-1}$ in the patient who died day 10 of either pulmonary embolus or myocardial infarct. It was unclear if his chemotherapy played a role in his sudden illness and renal dysfunction. The second patient had a rise from a baseline of 99 $\mu$mol l$^{-1}$ to 138 $\mu$mol l$^{-1}$ on day 1 of the sixth cycle of chemotherapy. The reason for this rise in serum creatinine late in the treatment course was unclear but it subsequently returned to normal and is presumed to have been treatment related. Four additional patients had their serum creatinine rise by more than 20% but the maximum serum creatinine did not exceed the upper limit of normal values. Two patients had mild hearing impairment: one complained of hearing loss but hearing was not formally tested; a second patient had mild intermittent tinnitus for cycles 1 and 2. Three patients reported mild paresthesiae in their distal extremities.

An analysis of response rate by known prognostic factors (Seifter & Ihde, 1988) including number of sites of tumour involvement, performance status, liver metastases and LDH level failed to reveal any significant differences.

Only four of the 14 patients who failed to respond to VP-16-carboplatin, were treated with second-line chemotherapy. Three received CAV chemotherapy and none responded. One was treated with VP-16-cisplatin and failed to respond and subsequent treatment with CAV was also ineffective.

Of the 18 responding patients, 12 have relapsed and seven received second-line chemotherapy. Six were treated with CAV: 2 are not evaluable, 3 had progressive disease and there was one partial response. Two patients were treated with VP-16-cisplatin (one after failing CAV): one patient progressed and the other responded but succumbed to pneumonia two months after his relapse from first-line treatment.

### Discussion

Although a small proportion of patients with limited small cell lung cancer may be cured with combined modality approaches in which chemotherapy is the central therapeutic modality, extensive disease remains an incurable disease with a median survival time of approximately 7 to 9 months (Greco et al., 1978). Current investigative strategies for extensive SCLC include assessment of new agents as first line therapy, regimens designed to more effectively utilize existing cytotoxic agents and efforts to minimize the toxicity of agents used as palliative therapy.

As indicated earlier, the combination of VP-16 and cisplatin is highly active in patients with newly diagnosed or relapsed disease. Data from a randomized trial by the NCIC clinical trials group indicate that the incorporation of this combination in an alternating schedule with standard CAV (cyclophosphamide, adriamycin, vincristine) improves survival when compared to the use of CAV alone (Evans et al.,

1987). However, that trial and others (Porter *et al.*, 1985; Evans *et al.*, 1985, 1986) documented that a small but significant number of patients experience important renal toxicity as a result of the cisplatin therapy. It would therefore be a useful addition to the oncologist's armamentarium to have an agent with less nephrotoxicity compared with the parent compound if antitumour activity was not reduced.

This multicentre study demonstrates that VP-16-carboplatin is both active and relatively non-toxic. However, the overall response rate of 56% in extensive disease (CR, 16%; PR 41%) appears inferior to the results reported for VP-16-cisplatin as first-line therapy. Evans reported a complete response rate of 29% and partial response rate of 58.5% in 17 selected extensive disease patients seen at several University of Toronto institutions (Evans *et al.*, 1985). When all studies reporting results of VP-16 and cisplatin as first-line therapy in extensive small cell lung were reviewed, the overall response rate was 83% with 29% of patients achieving CR and 54% a PR (Evans *et al.*, 1986).

Smith *et al.* have reported that 18 of 24 (75%) extensive disease patients had a partial response and 3 (13%) had a complete response in a single institution study using the same doses and schedule of VP-16 and carboplatin as reported in this study (Smith *et al.*, 1987). It was noted in their report that the median duration of response was short (5.5 months) with a predicted continuing remission rate at one year of $<10\%$. On the other hand, the median survival time was 9.5 months. Our results are similar with a median duration of response of 5.8 months and median survival time of 8.1 months. It should be noted that these survival times are comparable to those seen in the large National Cancer Institute of Canada multicenter trial in extensive small cell lung cancer (Evans *et al.*, 1987). In that study, the median survival times were 8.0 and 9.6 months for the standard (6 cycles of CAV) and alternating (CAV alternating with VP-16-cisplatin for 6 cycles) regimens respectively. In addition, the median survival time of extensive disease patients receiving VP-16-cisplatin as first-line therapy ranges from 5.8 to 9 months with a mean of approximately 7 months (Evans *et al.*, 1987). Although direct comparisons between these studies cannot be made because of potential differences in study populations, it is reassuring that the survival of patients treated with VP-16-carboplatin is not markedly different from that of other commonly employed strategies.

The results achieved by Smith *et al.* (1987) were achieved with a treatment plan of only four treatment cycles although the median number of treatment cycles actually given was not stated. We attempted to treat all responding patients with six treatment cycles; the median number of treatment cycles for the whole group was 4.5.

Bishop *et al.* (1987) have also investigated the VP-16-carboplatin combination in a different dose and schedule. Carboplatin $100\,mg\,m^{-2}$ was given on each of the three treatment days ($300\,mg\,m^{-2}$ per course) with VP-16 $120\,mg\,m^{-2}$ day 1, 2, and 3 ($360\,mg\,m^{-2}$ per course). Their study included 94 previously untreated patients, 59 of whom had extensive disease. An attempt was made to give a total of six treatment courses to all responding patients. Overall, a median of 5 courses of chemotherapy were given. Nine percent of the extensive disease patients achieved a complete response and 49% had a partial response. The median relapse-free survival was 7.9 months and overall survival for extensive disease patients was 8.3 months.

Although the response rates in extensive disease patients have ranged from 56.2% to 88% in these three studies, the overall survival has been similar (8.1, 8.3, 9.5) and comparable to other commonly used first-line therapies.

It is clear that this regimen has a lower level of toxicity than the VP-16-cisplatin combination. Only 2 of 34 (6%) patients had elevation of serum creatinine above the upper limit of normal. In one case, the creatinine elevation occurred at the time of pancytopenia and possible congestive heart failure and was probably not directly related to carboplatin administration. In the other case, the elevation in creatinine occurred at the time of the sixth treatment cycle and promptly returned to normal. Nausea and vomiting occurred infrequently. Although this, in part, was attributable to the aggressive use of combination antiemetic therapy, only 3 of 32 (9%) patients had sufficient gastrointestinal upset to necessitate intravenous fluid replacement. Three patients reported mild paresthesiae and two also complained of transient tinnitus and/or hearing loss, demonstrating that this platinum analog is not totally devoid of neurotoxic side effects.

Myelosuppression is the major dose limiting side effect of this combination. Furthermore, myelosuppression appears to be cumulative as demonstrated by the fact that the proportion of patients receiving 100% of planned treatment doses dropped to 60% by cycle 4. In addition, some patients had severe degrees of marrow suppression even after an initial dosage reduction. On the other hand, anaemia requiring transfusion was observed only once in this series, which appears to be much less frequent than with VP-16-cisplatin. In a series of 31 patients receiving VP-16 and cisplatin as first-line therapy, anaemia of $100\,g\,l^{-1}$ or less occurred in 64% and 32% required one or more blood transfusions (Evans *et al.*, 1985). Although the single agent data suggested that carboplatin might induce serious thrombocytopenia in this combination, thrombocytopenia was generally mild and not dose-limiting.

Of some concern to several investigators participating in our study was the four week treatment interval. Against a disease as kinetically aggressive as small cell lung cancer, this long interval between treatments, may permit tumour regrowth. Although we could not document any adverse effect of this treatment interval from the data forms, a few patients reported that tumour-related symptoms returned in the week prior to their scheduled day 28 treatment.

We conclude from our results and those of others that the VP-16-carboplatin combination is active against extensive small cell lung cancer and better tolerated than VP-16 combined with cisplatin. Although there may be small differences between the two regimens in terms of antitumour efficacy, it would take a very large randomized clinical trial to demonstrate what is probably a small difference.

The NCI-C Lung Group has recently recommended that CAV alternating with VP-16-cisplatin be accepted as the new standard for the treatment of extensive small cell lung cancer (Evans *et al.*, 1987). However, for older patients with underlying renal dysfunction or those who would be at risk from a prehydration fluid challenge, the substitution of carboplatin for cisplatin in this alternating regimen would seem a reasonable treatment strategy.

The authors acknowledge the contributions made to patient accrual and management of the following investigators: Drs J. Pater, B. Campling (Kingston Regional Cancer Centre), Dr J. Jolivet (Hopital Notre-Dame, Montreal), Drs J. Latrielle, F. Letendre (Hotel Dieu de Montreal), Dr M. Van Olm (Tom Baker Cancer Centre, Calgary). We also thank Mrs Chris Allen for her careful preparation of the manuscript.

### References

BISHOP, J.F., RAGHAVEN, D., STUART-HARRIS, R. *et al.* (1987). Carboplatin (CBDCA, JM-8) and VP 16-213 in previously untreated patients with small cell lung cancer. *J. Clin. Oncol.*, **5**, 1574.

CALVERT, A.H., HARLAND, S.J., NEWELL, D.R. & 9 others (1982). Early clinical studies with cis-diammine 1, 1-cyclobutane dicarboxylate platinum II. *Cancer Chemother. Pharmacol.*, **9**, 140.

CANETTA, R., ROZENCWEIG, M. & CARTER, S.K. (1985). Carboplatin: The clinical spectrum to date. *Cancer Treat. Rev.*, **12**, 125 (Suppl. A).

EGORIN, M.J., VAN ECHO, D.A., TIPPING, S.J. *et al.* (1984). Pharmacokinetics and dosage reductions of cis-diammine (1,1-cyclobutane decarboxylato) platinum in patients with impaired renal function. *Cancer Res.*, **44**, 5432.

EVANS, W.K., OSOBA, D., FELD, R., SHEPHERD, F.A., BAZOS, M.J. & DEBOER G. (1985). Etoposide (VP-16) and cisplatin: An effective treatment for relapse in small cell lung cancer. *J. Clin. Oncol.*, **3**, 65.

EVANS, W.K., OSOBA, D., SHEPHERD, F.A., FELD, R. & DANG, P. (1985). VP-16 and cisplatinum as first line therapy for small cell lung cancer. *J. Clin. Oncol.*, **3**(11), 1471.

EVANS, W.K., SHEPHERD, F.A., FELD, R. *et al.* (1986). First-line therapy with VP-16 and cisplatin for small cell lung cancer. *Semin. Oncol.*, **13**, (Suppl. 3), 17.

EVANS, W.K., FELD, R., MURRAY, N. *et al.* (1987). Superiority of alternating non-cross resistant chemotherapy in extensive small cell lung cancer: Results of a multicenter randomized National Cancer Institute of Canada clinical trial. *Ann. Int. Med.*, **107**, 451.

GRECO, F.A., EINHORN, L.H., RICHARDSON, R.L. & OLDHAM, R.K. (1978). Small cell lung cancer: progress and perspectives. *Semin. Oncol.*, **5**, 323.

PORTER, L.L., JOHNSON, D.H. & HAINSWORTH, J.D. (1985). Cisplatin and etoposide combination chemotherapy for refractory small cell carcinoma of the lung. *Cancer Treat. Rep.*, **69**(5), 479.

SCHABEL, F.M., TRADER, M.W., LASTER, W.K. *et al.* (1979). Cis-dichlorodiammine platinum (II): Combination chemotherapy and cross-resistance studies with tumours of mice. *Cancer Treat. Rep.*, **63**, 1459.

SEIFTER, E.J. & IHDE, D.C. (1988). Small cell lung cancer: A distinct clinicopathologic entity. In *Lung Cancer: A Comprehensive Treatise*, Bitran *et al.* (eds) p. 270. Grune & Stratton Inc.: Orlando, Fla.

SIEROCKI, J.S., HILARIS, B.S., HOPFAN, S. *et al.* (1979). Cis-diamminedichloroplatinum (II) and VP-16-213: An active induction regimen for small cell carcinoma of the lung. *Cancer Treat. Rep.*, **63**, 1593.

SMITH, I.E., HARLAND, S.J., ROBINSON, B.A. & 7 others (1985). Carboplatin: A very active new cisplatin analog in the treatment of small cell lung cancer. *Cancer Treat Rep.*, **69**, 43.

SMITH, I.E., EVANS, B.D., GORE, M.E. & 4 others (1987). Carboplatin (paraplatin; JM8) and etoposide (VP-16) as first-line combination therapy for small cell lung cancer. *J. Clin. Oncol.*, **5**(2), 185.

VON HOFF, D.D. & ELSON, D. (1980). Clinical results with cisplatin in lung cancer. In *Cisplatin Current Status and New Developments*, Vol. 1, Prestayko *et al.* (eds) p. 445. Academic Press: Orlando, Fla.

WOODS, R.L. & LEVI, J.A. (1984). Chemotherapy for small cell lung cancer (SCLC): A randomized study of maintenance therapy with cyclophosphamide, adriamycin and vincristine (CAV) after remission induction with cisplatinum, VP-16-213 and radiotherapy. *Proc. Am. Soc. Clin. Oncol.*, **3**, 214 (*abstr. C-836*).